# Bioaccessibility and Functional Food Potential of *Equisetum telmateia* Ehrh. Against Diabetes-Induced Kidney Disorders

**DOI:** 10.3390/foods13244092

**Published:** 2024-12-18

**Authors:** Timur Hakan Barak, İnci Kurt-Celep, Engin Celep

**Affiliations:** 1 Department of Pharmacognosy, Faculty of Pharmacy, Acibadem Mehmet Ali Aydınlar University, Ataşehir, İstanbul 34752, Türkiye; engin.celep@acibadem.edu.tr; 2 Department of Biotechnology, Faculty of Pharmacy, İstanbul Okan University, Tuzla, İstanbul 34940, Türkiye; inci.celep@okan.edu.tr

**Keywords:** *Equisetum telmateia*, LC-MS/MS, in vitro digestion, anti-diabetic, diuretic

## Abstract

Various species from the genus *Equisetum* are recorded as food and folk medicine against both kidney complications and diabetes. *Equisetum telmateia* Ehrh. is documented as a folk remedy in Türkiye against several kidney disorders. This study was designed to evaluate the possible protective mechanisms of *E. telmateia* EtOH extract (ETE) against kidney disorders and diabetes through different routes, such as the prevention of ROS formation, inhibitory potential against various DM-related enzymes, and a reduction in the amount of the mediators leading to disorders in both systems at the cellular level. The objective was to achieve advanced precision for in vitro results while considering the effect of GIS on oral consumption. Both phytochemical and bioactivity studies were conducted before and after simulated digestion. The results showed that ETE is a rich source of flavonoids and phenolic acids. In addition, it has significant antioxidant and enzyme inhibitory potential. Treatment also yielded promising results at the cellular level for both antioxidative and inhibitor proteins, which may play a role in the pathogenesis of kidney disorders and diabetes. Following the in vitro digestion procedure, both the number of phytochemical ingredients and bioactivity parameters showed a considerable decreasing trend; however, the results are still significant enough to justify the traditional utilization of the genus *Equisetum*. This investigation demonstrated that ETE has noteworthy potential as a functional food for protection against diabetic kidney disease.

## 1. Introduction

The single genus in the family Equisetaceae, also known as horsetail in English and as atkuyrugu or kirkkilit in Turkish, is *Equisetum* L. There are seven species in the Turkish flora, compared to about 32 identified species worldwide. They are typically perennial plants with silica-rich cell walls that thrive in humid and damp environments. *Equisetum telmateia* Ehrh. is widely distributed in continents such as Europe, West Asia, Northwest Africa, and North America [1]. Various studies have explored the potential of the *Equisetum* genus as a promising source of functional foods due to its diverse bioactive compounds [2]. Furthermore, extracts prepared from different *Equisetum* species have been proposed as valuable ingredients for nutraceutical formulations owing to their potential health benefits [3]. However, certain species, including *E. telmateia*, still require further investigation to assess their full potential as functional food sources. In addition, all species of *Equisetum* are important biological remedies in Turkish folk medicine against various diseases. Among these species, *E. telmateia* has an important place in Turkish folk medicine. It is used in Anatolia for various diseases such as cough, hemorrhoids, and asthma; to treat stomach pain or peptic ulcers; in the case of eczema, acne, and rheumatism; against the pain of fractures; to strengthen hair, skin, and nails against oral infections; against fungal and parasitic infections; and against heart failure and atherosclerosis. Together with other utilizations, *E. telmateia* is prominent as an ailment against urinary disorders. Ethnobotanical records showed that it is used as a diuretic, thus being used to pass kidney stones or sand, and against pyelonephritis, prostatic hypertrophy, and cystitis [4]. Previous studies demonstrated that species from the genus *Equisetum* are significantly rich in phenolic acids and flavonoids. It is known that these compounds are responsible for the potent antioxidative effects of numerous plants. In addition, plants that possess high contents of these compounds are known for their anti-inflammatory and anti-diabetic properties. Hence, several species from the genus *Equisetum* are recorded as anti-diabetic ailments in traditional medical systems, and various scientific studies have confirmed such anti-diabetic uses [5]. However, *E. telmateia* has not been investigated in that manner, to our knowledge.

In vitro studies are reliable, low-cost, and reproducible; thus, they are widespread in scientific investigations and serve as valuable starting points for the systemic assessment of a variety of bioactivities of plant extracts. However, the direct application of plant extracts in in vitro assays neglects the pharmacokinetic effects induced by the gastrointestinal system and overlooks the bioavailability phenomenon. Previous studies showed that these effects may lead to significant alterations in bioactivity. This outcome takes place due to significant modifications of phytochemical ingredients after the application of the simulated digestion process [6]. 

Diabetes is the most prevalent metabolic disease, affecting hundreds of millions of patients worldwide [7]. In addition, kidney problems are among the most widespread manifestations of diabetes. Various studies have shown that hyperglycemia-induced advanced glycation end product (AGE) formation plays a central role in the pathogenesis of diabetes-related kidney disorders. AGE accumulation on receptors (RAGE) is known to generate oxidative stress with increased ROS formation and inflammation, resulting in deleterious effects on diabetic conditions [8]. In addition, previous studies reported that increased ROS in the target tissues lead to the activation of NF-kB and AP-1, which are both involved in the pathogenesis of diabetic nephropathy [9]. Moreover, diuretics are commonly used in kidney disorders, and studies have shown that they may be beneficial in DM-related kidney disorders [10]. Thus, investigating herbal medicines for preventing ROS formation, inhibiting enzymes related to diuresis, and acting as molecular triggers in the pathway for diabetic kidney damage may provide accurate knowledge to verify their ethnobotanical utilizations. 

In light of this information, the anti-diabetic and diuretic properties of ethanolic extracts from the aerial parts of *E. telmateia* were investigated using several in vitro assays. To obtain better accuracy, an in vitro human digestion simulation procedure was employed on the extract prior to chromatographic analysis and bioactivity studies. Even though in vivo assays may yield more accurate outcomes, economic and ethical considerations have increased the importance of the alternative methods. It is known that the bioactivity of plant extracts is based on their phytochemical ingredients. Thus, extensive LC-MS/MS analysis was employed on the extract and fractions obtained via in vitro gastric simulation. For the evaluation of the in vitro antioxidant potential, FRAP, TOAC, and DPPH assays were conducted on all fractions. In addition, α-amylase, α-glucosidase, PTP1B, advanced glycation end product (AGE), and urease inhibition assays were conducted on all fractions to evaluate the anti-diabetic and diuretic properties and variations after digestion simulation. Moreover, cell culture studies were employed to determine *Equisetum telmateia* ethanolic extract (ETEBFR) and its bioavailable fraction (ETEIN) on HEPG2 cells in order to elucidate its possible mechanism of action at the cellular level. A WST-1 assay was performed to determine the non-toxic concentration, and a DCFDA assay was conducted to determine the antioxidant capacity of the fractions at a cellular level. Furthermore, Western blot analysis was employed to determine the levels of important proteins involved in the apoptosis pathway, including phosphorus-NfκB and RAGE in the HEPG2 cell line, which are all induced with diabetes and kidney disorders.

## 2. Materials and Methods

### 2.1. Plant Material

Aerial parts of *Equisetum telmateia* Ehrh. were collected from Demirköy/Kırklareli by Dr. Engin Celep in September 2022. Botanical identification was completed by Dr. Ahmet Doğan, and the voucher specimens were added to the Marmara University Faculty of Pharmacy Herbarium (MARE 23084). Then, the plant material was dried and powdered before extraction. Next, 700 mL of 96% EtOH was added to 50 g of the powder and macerated on a shaker for 24 h. After that, the remaining EtOH was filtered through filter paper and EtOH was added on the remains. This procedure was repeated three times. Afterwards, the filtered parts were combined, and the EtOH was evaporated with a rotary evaporator and then lyophilized. At the end of the extraction, the dry extract (2.6 g 5.2%, yield) was weighed. The dry extract was stored at −20 °C until further analysis. All chemicals, enzymes and references used in the experiment protocols were purchased from Sigma Chemical Co. (St. Louis, MO, USA).

### 2.2. In Vitro Digestion Simulation Procedure

As mentioned previously, a human GI digestion simulation model was used for this study [11]. After the sample solution was added to 20 mL of the gastric environment, the mixture was incubated for two hours in a shaking water bath to simulate peristaltic movement. To represent the post-gastric (PG) sample, the mixture was set aside. The content that diffused into the dialysis membrane (i.e., modeling the absorption process) was named IN after two hours of incubation in intestinal medium with cellulose dialysis tubing and a sufficient amount of powdered NaHCO_3_.

### 2.3. Phytochemical Profiling and LC-MS/MS Analysis

Fifty-three phytochemicals containing various phenolic compounds were analyzed according to a previously validated method using LC-MS/MS for all fractions [12]. A Nexera model UHPLC (Shimadzu) combined with tandem MS equipment was used to perform the quantitative investigations. Shimadzu Lab Solutions software (https://www.shimadzu.com/an/products/software-informatics/labsolutions-series/index.html accessed on 24 October 2024) was used to process the LC-ESI-MS/MS results. The column temperature was set to 40 °C. The elution gradient was composed of eluent A (water, 5 mM ammonium formate, and 0.1% formic acid) and eluent B (methanol, 5 mM ammonium formate, and 0.1% formic acid). The gradient elution profiles used were 20–100% B (0–25 min), 100% B (25–35 min), and 20% B (35–45 min). In addition, the solvent flow rate and injection volume were determined to be 0.5 mL/min and 5 µL, respectively.

### 2.4. In Vitro Phenolic and Antioxidant Profiling

The total phenolic and flavonoid contents of the samples were evaluated according to a previously described method. Folin–Ciocalteu reagent (FCR) was used, and the absorbance at 765 nm was measured to determine the total phenol content of all the fractions. After the incubation period, the total flavonoid content was measured at 415 nm with a previously described method [13]. Antioxidant capacity assays were carried out as previously explained [14]. Butylated hydroxytoluene (BHT) was used as the reference compound for DPPH radical scavenging activity, and the results are given as BHT equivalents. To determine the cupric reducing antioxidant capacity (CUPRAC), ascorbic acid was used as a reference compound, and the absorbance was measured with a 450 nm spectrophotometer. Ascorbic acid was similarly used as the reference antioxidant compound in the total antioxidant capacity (TOAC) assay. Following a 90-min incubation period, absorbance was measured at 695 nm. The ferric reducing antioxidant power (FRAP) was determined via a redox-linked colorimetric method, and the absorbance of the mixture was read at 593 nm.

### 2.5. Enzyme Inhibition Assays

Urease: The optimized protocol in the literature was used to detect the inhibitory effects of 1 mg/mL of the ETBFR, ETEIN, and ETEPG fractions on the urease enzyme. The sample group was obtained by mixing 100 μL of BFR, IN, and PG fractions at a concentration of 1 mg/mL with 15 μL of urea enzyme and 850 μL of 0.1 M urine. Unlike the sample groups, the blanks contained 0.1 M phosphate buffer (at pH 7.4) instead of the same volume of enzyme, while the control groups did not contain plant samples. A reference concentration of thiourea ranging from 62.5 to 1000 μg/μL was used in the experiment. After all the experimental groups were established, the samples were incubated for 60 min at 37 °C for pre-incubation. Then, 500 μL of solution A (50 mL of phenol and 2.5 g of sodium nitroprusside, prepared in distilled water) and 500 μL of solution B (50 mL of 250 mg sodium hydroxide and 820 μL of 5% sodium chloride) were added and kept for another 30 min at 37 °C, followed by an absorbance measurement at 640 nm [15].

α-Amylase: The α-amylase inhibitory activity was measured according to the method previously described by Inan et al. with slight modifications [16]. Twenty microliters of BFR, IN, and PG samples, obtained from the ETE and subjected to the gastric simulation method, and 10 µL of α-amylase (2 units/mL) in buffer solution were added to each well and allowed to incubate for 45 min at 37 °C. Then, 20 µL of starch solution (1% in water (*w*/*v*)) was added, and the mixture was incubated again. At the end of the incubation period, 3,5-dinitrosalicylic acid (DNS) solution was added, and the mixture was boiled at 90 °C for 20 min. The absorbance at 540 nm was read using a 96-well microplate reader. Acarbose was used as a positive control. The negative control was performed without adding the sample. The α-amylase inhibitory activity is expressed as the percentage of inhibition.

α-Glucosidase: The α-glucosidase inhibitory activity was determined according to a previously described procedure with minor modifications [16]. In each well of a 96-well plate, 20 µL of α-glucosidase and 20 µL of BFR, IN, and PG samples were placed in phosphate buffer and incubated for 15 min at 37 °C. Then, 20 µL of substrate (2.5 mM 4-nitrophenyl α-D-glucopyranoside) was added and allowed to incubate again. Next, 0.2 M Na_2_CO_3_ was added to terminate the reaction and the absorbance was read at 405 nm using a Thermo Scientific™ Varioskan 96-well microplate reader. Quercetin was used as a positive control. The negative control was performed without adding ETE.

PTP1B: PTP1B has a negative regulatory effect on insulin signaling through the phosphorylation of thyroxine residues in the insulin receptor downstream pathway. For this reason, a literature-presented and optimized experimental protocol was followed to detect the inhibitory effect of ETE on PTP1B. Using ursolic acid as a positive control in the experiment, the effect of ETE on the inhibition of PTP1B by the BFR, IN, and PG fractions obtained after a gastric simulation was calculated [17].

### 2.6. Advanced Glycation End Products (AGEs)

The glycated bovine serum albumin (BSA) formation method (with a minor modification) was conducted as previously described. In brief, BSA (10 mg/mL) was incubated with 0.5 M glucose in 0.1 M phosphate-buffered saline (PBS) containing sodium azide in the dark at 37 °C for 40 h. Before incubation, the gastrointestinal digestion products (BFR, IN, and PG) of ETE were added to the mixtures. Quercetin was used as a positive control. Glycose-BSA formation, also known as AGE formation, was measured in a Thermo Scientific™ Varioskan™ Lux multimode microplate reader using fluorescence intensity (FI) at a 370 nm excitation wavelength and a 450 nm emission wavelength After measurement, the previously given equation was used to calculate the AGE inhibition activity at different concentrations [18]. 

### 2.7. Cell Culture

The hepatocellular cell line HepG2 was selected as the target cell line for the insulin resistance (IR) model for the investigation of molecular pathways involved in diabetes metabolism. HepG2 cells were cultured for increasing durations (6, 12, 18, 24, 32, and 48 h) in a low-glucose medium supplemented with increasing insulin concentrations (0.005, 0.05, 0.5, 5, and 50 µmol) at various concentrations determined in previous studies on the same cell line. A commercially available HepG2 hepatocellular carcinoma cell line was purchased from the American Tissue Culture Collection (ATTC). Then, an IR model was created by treating the cells with 50 µmol of insulin every 24 h for insulin resistance. After developing IR in HepG2 cells, 20 µg/mL metformin was used as a positive control [19,20].

### 2.8. WST-1 Assay

After the formation of insulin resistance in commercial HepG2 cells supplied by ATCC, as described in Section 2.7, the WST-1 test was used to determine the non-toxic concentrations of ETEBFR and ETEIN in these cells. WST is a technique in which a color change occurs depending on the vitality of the cells; in other words, it depends on metabolic activity and can be measured spectroscopically. The cells were plated in a 96-well plate at 10,000 cells/well. The next day, increasing doses of ETEBFR and ETEIN were applied to the HepG2-IR cells for 24, 48, and 72 h. The control group consisted of HepG2-IR cells not treated with ETEBFR or ETEIN, and the cell viability was calculated at 100% confluency at all time points compared to the control group [21].

### 2.9. Western Blot Analysis

ETEBR and ETEIN were administered at 125 µg/mL separately for 48 h to insulin-resistant (IR) HepG2-IR cells to investigate the molecular pathways involved in diabetes, followed by variable metabolic syntheses of the phospho-NFκB p65, RAGE, and AP-1 proteins via the Western blot protocol previously described in the literature. β-actin was used as a loading control to ensure that the molecular weight of the proteins transferred to the membrane of 15% nitrocellulose was loaded with the same volume of cell lysates. The membranes were incubated overnight with the target antibodies. Then, the membranes were incubated with a horseradish peroxidase-conjugated secondary antibody at room temperature for two hours. After the incubation, a commercially available enhanced chemiluminescence (ECL) solution, known as a Western blotting detection reagent, was diluted 1:10 and photographed using a ChemiDOC device after 1 min of treatment with the membrane. The band densities of each protein were compared to beta-actin, and quantitative analysis was carried out using the ImageJ program [22]. 

### 2.10. DCFDA Assay

Following the development of the IR model in HepG2 cells, reactive oxygen species (ROS), which cause oxidative stress in liver cells, reached high levels and caused mitochondrial dysfunction. For this reason, the DCFDA method described previously was applied to detect the increased amount of ROS in HepG2-IR cells and the changing ROS levels in the cells after the application of ETEBFR and ETEIN [22].

### 2.11. Statistical Analysis

The experiments were performed at least in triplicate. The results are presented as the means ± standard deviations. Statistical comparisons were made using one-way analysis of variance (ANOVA) for the enzyme inhibition experiments, followed by 2-way ANOVA with a multiple comparisons test for the WST-1, Western blot, and DCFDA assays. A statistically significant difference was defined as *p* < 0.05.

## 3. Results and Discussion

### 3.1. Phytochemical Evaluation

**Figure 1:**
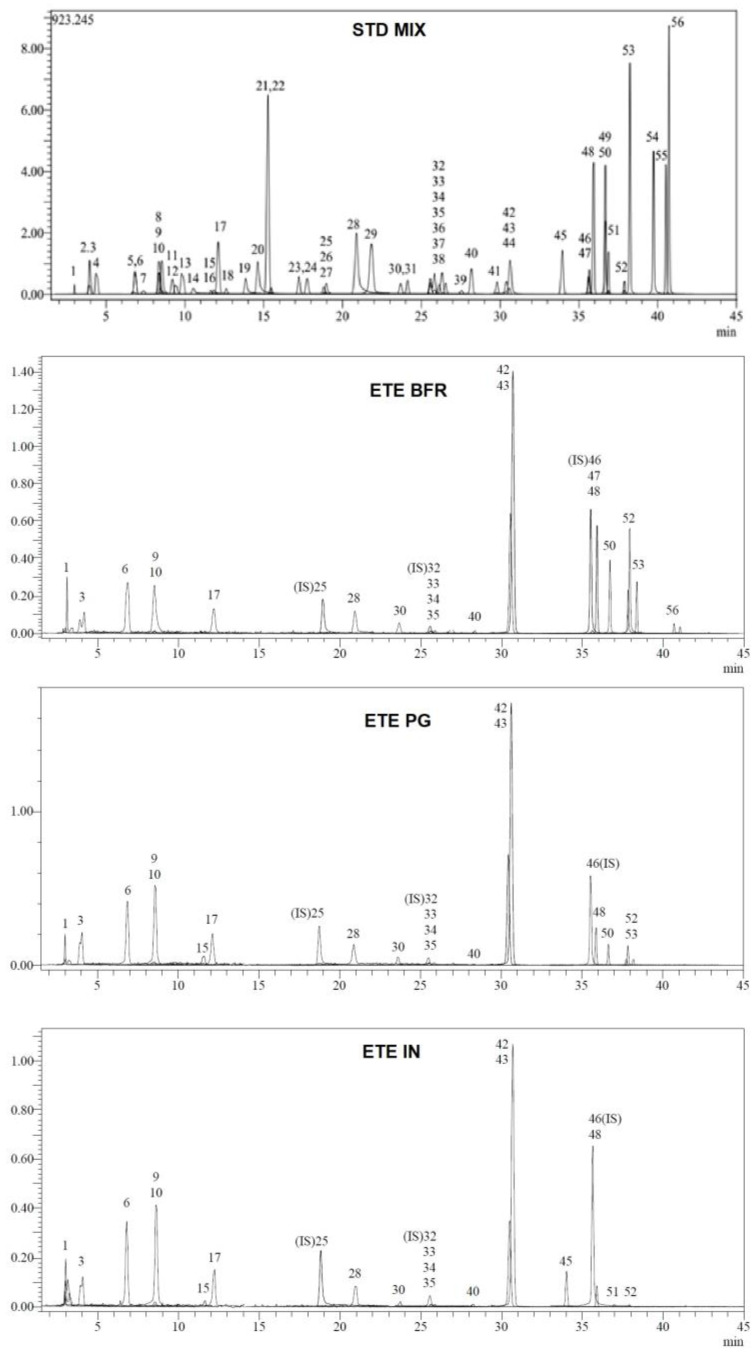
LC/MS/MS chromatogram of the samples and the mixture of standards.

A validated LC-MS/MS method was used for detailed qualitative and quantitative analyses of all fractions of the ETE. In this method, 53 different compounds were analyzed, with 21 different compounds detected in at least one of the fractions and 6 of them quantified in significant amounts (Table 1 and Figure 1). Kaempferol and its glycosides were detected as the major flavonoids in the main extract before digestion; the quantified amounts were 7.02 µg/g for kaempferol-3-O-rutinoside, 3.11 µg/g for kaempferol-3-O-glucoside, and 1.29 µg/g for kaempferol in its aglycon form. These findings correspond to our previous findings. Milovanovic et al. quantified flavonoid glycosides in five different species of *Equisetum* from Serbia and reported that *E. telmateia* has one of the richest contents of kaempferol and its glycosides [23]. Moreover, there are other reports demonstrating high amounts of kaempferol and its glycosides in *E. telmateia* from different regions [24,25]. However, after simulated digestion, the bioavailability of these flavonoids was relatively low. Kaempferol slightly decreased in the IN fraction and the amounts of kaempferol-3-O-rutinoside and kaempferol-3-O-glucoside significantly decreased. Parallel outcomes were observed in previous studies. Feng et al. showed that the kaempferol-3-O-glucoside content of *Nelumbo nucifera* Gaertn. leaves remained unchanged after gastric digestion but was significantly reduced after simulated intestinal digestion [26]. In addition, Bortolini et al. reported that the kaempferol content of Nastrutium decreased following a digestion simulation with zero bioavailability [27]. Protocatechuic acid and protocatechuic aldehyde were observed in significant amounts from the BFR fraction of the ETE (1.17 and 0.68 µg/g, respectively). In previous studies, significant protocatechuic acid contents were detected in *E. telmateia* extracts. Nunes et al. quantified 2.85 ± 0.41 mg/100 g dry weight protocatechuic acid from samples collected in Portugal, and Yeganegi et al. detected significant amounts of this acid in samples from Iran [24,28]. In contrast to kaempferol derivates, these phenolic compounds have shown resistance to in vitro gastric simulation and were quantified in equivalent amounts in the PG and IN fractions. Previous studies showed intricate results on the effects of in vitro digestion on protocatechuic acid content and its bioavailability. Hilary et al. investigated the effects of in vitro digestion on phenolic composition and bioaccessibility in different preparations from *Phoenix dactylifera* seeds [29]. The results showed conflicting alterations in protocatechuic acid content, with a significant increment detected in the protocatechuic acid content in seed extracts, while a strong reduction was seen in seed bread. On the contrary, equivalent amounts were measured for seed powder. An interpretation of this can be that the bioaccessibility and durability of phenolic ingredients are highly dependent on complex interactions between the ingredients of the extracts and the unique characteristics of plant parts. Furthermore, epicatechin was found to be absent in the BFR fraction, while after the gastric digestion procedure, a significant amount was measured in the PG fraction; it was also detected in lower amounts in the bioaccessible phase (5.63 and 1.13 µg/g, respectively). It has been shown in the literature that *E. telmateia* is rich in proanthocyanidins [25], and these compounds consist of condensations of catechins, including epicatechin [30]. The abrupt occurrence of epicatechin in the PG phase together with its absence in the extract may result from the disruptive gastric environment with a low pH, high temperature, and enzymes leading to the disintegration of proanthocyanidins into their monomers, such as epicatechin. These rich phenolic profiles and their alterations after the simulated digestion are expected to immensely influence the bioactivities of the extract. The same alterations were also observed in the total phenol and flavonoid content assays. According to the results of the in vitro evaluation, the total phenolic and flavonoid amounts were decreased significantly after the simulated digestion and showed lower bioavailability (Table 2). 

### 3.2. Evaluation of Antioxidant Capacity

It is well established that elevated oxidation with oxidative stress leads to increased ROS formation, which plays an indisputable role in the pathogenesis of various conditions, including diabetes and kidney disorders [31]. Thus, revealing the antioxidative properties of herbal extracts may provide key information about their possible role in preventing chronic complications. For this reason, several in vitro and cell culture assays were conducted in this study to determine the antioxidant capacity of the ETE before and after simulated digestion. Similarly, numerous studies have been carried out on the antioxidant potential of *Equisetum* species, including *E. telmateia* [5]. Stajner et al. investigated the antioxidant potential of three different species of *Equisetum* from Serbia using several different assays, including FRAP and DPPH, and their results showed that *E. telmateia* extracts showed the greatest potential among all the studied extracts [32,33]. Likewise, other studies have shown the significant antioxidant potential of different in vitro methods in *E. telmateia* [24,28,34]. Even though the antioxidant potential of *E. telmateia* has been shown through various in vitro studies, none of these studies considered the effect of the gastrointestinal tract on the extracts or the bioavailability of the active ingredients. Hence, in the current study, in vitro assays such as DPPH, TOAC, FRAP, and CUPRAC were conducted on fractions of ETE considering the effects of the GI tract and bioaccessibility. The results given in Table 3 show that the ETE showed significant in vitro antioxidant potential prior to digestion; however, the bioactivity decreased significantly through the steps of the digestion simulation. DPPH radical scavenging activity was measured as 272 ± 1 mg BHTE/g dry extract, but it was significantly reduced after gastric digestion in the bioaccessible fraction. The same results were observed in the TOAC, FRAP, and CUPRAC assays: after gastric and intestinal digestion, TOAC decreased from 853 ± 15 to 329 ± 18, FRAP decreased from 1.83 ± 0.14 to 0.38 ± 0.03, and CUPRAC from 387 ± 12 to 86.6 ± 1.5 in the bioavailable fraction.

These results are analogous to the alterations in the phytochemical profile, where similar reductions were observed in terms of phenolics. As expected, phenolics are responsible for the antioxidant potential of ETE, and decreases in their amounts also led to a reduction in the bioavailability of the extract. 

**Figure 2:**
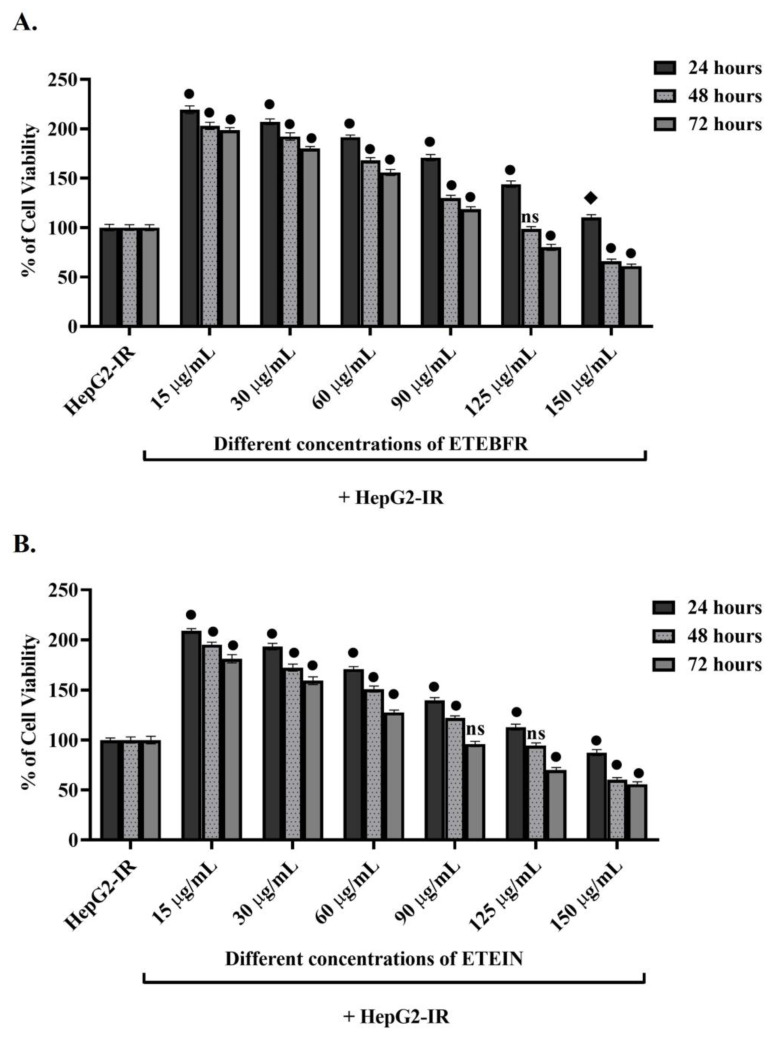
Determination of non-toxic concentrations of ETEBFR and ETEIN extracts in insulin-resistant HepG2 (HepG2-IR) cells by WST-1. (**A**) The % value of cell viability with increasing concentrations of ETEBFR applied to HepG2-IR cells for 24, 48, and 72 h. (**B**) The % value of cell viability with increasing concentrations of ETEIN applied to HepG2-IR cells for 24, 48, and 72 h. HepG2-IR cells were used as the control cell group and analyzed using 2-way ANOVA with multiple comparisons in the GraphPad Prism program. ● Statistically significant value at *p* ≤ 0.0001; ♦ statistically significant at *p* ≤ 0.001. ns = statistically similar results (not significant).

For better accuracy, the current study also investigated the antioxidant potential of ETE at the cellular level with the DCFDA assay, a frequently used method because of its specific ROS staining properties. HepG2 cells treated with H_2_O_2_ for extensive ROS formation were used for the assay, and the reduction in fluorescence in the cell culture after 24 and 48 h was measured in comparison to metformin, which is a standard anti-diabetic agent; the results are given in Figure 2. A high fluorescence intensity was measured in the non-treated cells at both time points. The standard substance, ETEBFR, and ETEIN significantly reduced the fluorescence, indicating antioxidant bioactivity at the cellular level. ETEBFR at a dose of 125 µg/mL had weaker bioactivity than did metformin at 20 µg/mL. However, the bioaccessible fraction showed a lower yet significant antioxidant capacity at both time points. These observations are consistent with the alterations in the phytochemical profile and in vitro antioxidant capacities of the fractions. The DCFDA assay has been used to determine the cellular antioxidant levels of various herbal medicines [22]. Although this is the first study to investigate the cellular antioxidant capacity of *E. telmateia* to our knowledge, several previous studies have investigated the differentiation of the cellular antioxidant capacities of herbal extracts before and after simulated digestion. Ge et al. investigated the antioxidant activity of an ethanol extract from Ligusticum after being subjected to digestion simulation [35]. A significant reduction in the cellular antioxidant level was reported after digestion simulation, which is consistent with our findings. In light of these results, it was established that ETE had significant antioxidant capacity, even though this potential decreased after digestion, and the bioaccessible fraction still showed significant antioxidant bioactivity in both in vitro studies and at the cellular level when compared with the results from the literature.

### 3.3. In Vitro Enzyme and AGE Inhibition Studies

There are various ethnobotanical records of the effects of several species from the *Equisetum* genus on kidney disorders and their diuretic potential. In addition, *Equisetum telmateia* is used for treating kidney disorders such as kidney stones, nephritis, and urinary tract infections in folk medicine in various countries, including Türkiye, Spain, Italy, and Bosnia [5]. It is well known that diuretics may be beneficial for treating kidney disorders, especially kidney stones. Moreover, diuretic utilization of *E. telmateia* in traditional medicine in Türkiye has also been recorded [36]. Thus, an anti-urease inhibition assay was conducted on ETE to determine the potential diuretic properties of the extract. It was previously shown that different extracts of *E. telmateia* showed significant urease inhibition activity [37]. However, that study disregarded pharmacokinetic alterations of the extract after oral consumption. Hence, in this study, all the fractions of ETE were investigated along with in vitro digestion simulation for more accurate determination of the in vitro anti-urease inhibition potential, and the results are given in Table 4. The results show that ETE notably inhibited urease enzyme activity before digestion (92.5 ± 1.9%). However, a significant reduction was detected after the digestion simulation. Nonetheless, significant inhibition was still detected, which provides a meaningful foundation for traditional utilization. 

Diabetes is a widespread chronic disease that leads to various complications in the human body. Some of the most prevalent and malicious outcomes of diabetes are kidney disorders. Thus, diabetic control is essential for kidney homeostasis. The anti-diabetic potential of the genus *Equisetum* has been shown in various studies. In one study, a methanolic extract of *E. arvense* was studied on streptozotocin-induced diabetic male rats, and the results showed that blood glucose and urinary microalbumin and creatinine levels were reduced significantly [38]. Moreover, *E. giganteum* and *E. myriochaetum* showed significant anti-diabetic activity in vivo [39,40]. α-Amylase and α-glucosidase enzymes are important targets for diabetes control due to their significance in postprandial diabetes [41]. Likewise, PTP1B is an important target enzyme in the control of diabetes, which is a negative regulator of insulin pathway signaling and is deliberated as a likely potential therapeutic target [42]. For the above reasons, the inhibition potential of ETE against α-amylase, α-glucosidase, and PTP1B enzymes was investigated in the present study. Previous studies showed that another *Equisetum* species, namely *E. arvense*, showed significant potential for inhibiting α-amylase and α-glucosidase enzymes [43]. This is the first study investigating the α-amylase and α-glucosidase enzyme inhibition potential of *E. telmateia* and the PTP1B inhibitory activity of a species in the *Equisetum* genus to our knowledge. The results from Table 4 show that ETE had significant inhibitory activity against the three aforementioned enzymes before the simulated digestion. Although a significant decrease was observed after the in vitro simulation process, noteworthy inhibitory activity was still detected in both the PG and IN phases. These reductions are consistent with the alterations in the phenolic profile and antioxidant activity. It is well known that phenolic ingredients are generally considered as active secondary metabolites of plant extracts due to their antioxidant and enzyme inhibitory bioactivities [44]. Thus, slight reductions may be explained via these phytochemical alterations.

Advanced glycation end products (AGEs) are formed non-enzymatically between carbonyl groups of sugars and amino groups of some macromolecules. The most important harmful effect of AGEs is the generation of oxidative stress and aggregation of inflammation. AGEs are associated with various pathological conditions such as diabetes and its complications such as diabetic kidney disease [8]. Thus, inhibiting AGE formation is a valuable target to preventing the damage of hyperglycemia on kidney health. Accordingly, in this study, the AGE-inhibiting potential of the ETE before and after in vitro digestion was investigated. The results demonstrated that the ETE had a notable AGE inhibition ability, similar to the standard substance quercetin (Table 4). Corresponding to former results, this activity slightly diminished yet was still noteworthy following the digestion procedure. When the results of the in vitro inhibition studies are evaluated altogether, ETE appears to have valuable in vitro potential for utilization against diabetes and related kidney problems, which is in agreement with ethnopharmacological records.

### 3.4. Determination of Non-Toxic ETE Concentration

**Figure 3:**
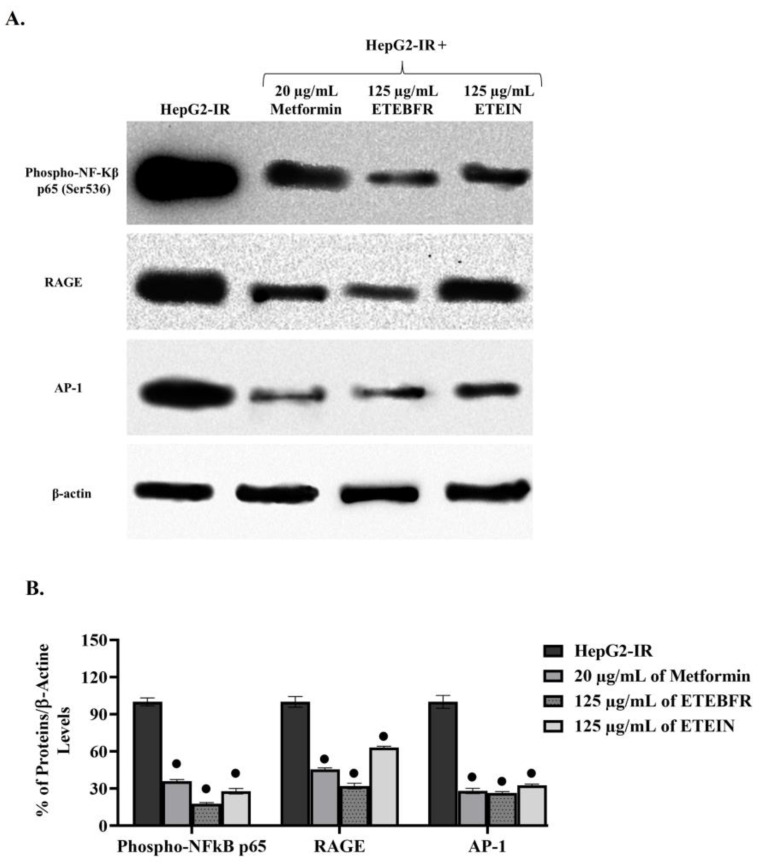
Detection of the changing protein levels in the molecular mechanism following the application of ETEBFR and ETEIN extracts at a non-toxic concentration of 125 µg/mL to HepG2-IR cells by Western blot. (**A**) Membrane image showing expression of phospho-NfκB, RAGE, and AP-1 proteins in the cellular pathway of HepG2-IR cells treated with 20 µg/mL of metformin (positive control), 125 µg/mL of ETEBFR, and 125 µg/mL of ETEIN extracts. (**B**) Statistical analysis of each protein compared to beta-actin, which has its own loading control. ● Significant at *p* ≤ 0.0001.

The WST-1 test (Figure 3) was performed to detect the non-toxic concentrations of ETEBFR and ETEIN sub-extracts obtained after gastric simulation via the ETE method was applied to HepG2-IR model cells. HepG2-IR cells were treated with ETEBFR and ETEIN sub-extracts for 24, 48, and 78 h. Metformin at 20 µg/mL, which is used to overcome insulin resistance and was previously optimized in HepG2-IR cells, was used as a positive control [20]. When the non-toxic concentrations of ETEBFR and ETEIN in Figure 3 were compared to the untreated HepG2-IR control cells, the viability of the cells appeared to decrease at an acceptable rate due to the increase in concentration at each time point. As shown in Figure 3A, ETEBFR at 150 μg/mL increased the cellular vitality by 10.35% at the end of the 24th hour, which then decreased to 66% after 48 h and to 61.04% after the 72nd hour. As shown in Figure 3B, these values were 87.5%, 60.43%, and 56%, respectively. A non-toxic concentration was determined for progressive experiments with a concentration-dependent decrease in the vitality rate of not less than 60% in the cells. In this case, 125 μg/mL was selected as a promising concentration for both ETEBFR and ETEIN. *Equisetum* species, which are commonly used in folk medicine, have been found to be free of cytotoxic effects [45]. The antimicrobial activity and toxicity of *Equisetum hyemale* extracts have been shown both in vitro and in vivo [5]. Several studies in the literature support our findings, as shown in Figure 3. In a study by Alavarce et al., no toxic effects were observed as a result of increased concentrations of extracts obtained from aerial parts of *E. giganteum*, both in human palatine epithelial cells and in human monocyte cells [46]. Similar results were obtained in another study involving a clinical trial of *Equisetum bogotense*, in which 0.75 g of herbal infusion was administered daily to 25 people and no side effects were revealed other than toxicity [47]. Our findings, which are consistent with these data in the literature, suggest that ETE is a promising extract for the treatment of insulin resistance without causing cytotoxicity at the end of gastric simulation and may be beneficial for kidney health.

### 3.5. Determination of Alterations in Protein Synthesis via Western Blot

After the application of 125 µg/mL ETEBFR and ETEIN to HepG2-IR cells, Western blotting (Figure 3) was performed to determine the changes in phospho-NFκB, RAGE, and AP-1 protein synthesis in these cells. After the experiments were independently repeated three times, the proteins were quantitatively analyzed using the β-actin ratio, which is the loading control of each protein. Phospho-NFκB was reduced by 35.88% in metformin-treated cells compared to HepG2-IR control cells, and similar decreases were found for RAGE (45%) and AP-1 (28.06). On the other hand, after ETEBFR application, phospho-NFkB protein levels decreased by 18%, RAGE levels decreased by 32.06%, and AP-1 protein decreased by 27%. Similar results were obtained following the application of BFRIN to HepG2-IR cells: phospho-NfκB decreased by 28%, RAGE by 63.07%, and AP-1 by 32.55%. It is well known that creating insulin resistance in HepG2 cells causes instability and the accumulation of cellular reactive oxygen species (ROS) [48]. An increase in ROS leads to the development of many metabolic disorders, especially inflammation, in cells. In addition, ROS are an insidious factor underlying the pathogenic effects of insulin resistance, hyperglycemia, and type 2 diabetes. Phospho-NfκB, RAGE, and AP-1 proteins play crucial roles in regulating this molecular mechanism and restoring healthy insulin metabolism [49]. Studies have shown that NfκB is inactive in balanced metabolic pathways and under normal conditions, depending on the presence of IKB in the cytosol. However, NfκB is translocated to the nucleus and transformed into phospho-NfκB due to the phosphorylation of IKB in cases of inflammation in cells, increased ROS levels, the deterioration of insulin metabolism, type 2 diabetes, atherosclerosis, and many other metabolic disorders [50]. As a result of the accumulation of phospho-NF-κB, the active form of phospho-NFκB, in the nucleus, it has been found that it especially impairs the function of pancreatic β-cells and increases the production of lipotoxic products such as ceramide and diacylglycerol, which damage vascular reactivity and mitochondrial metabolism and cause obesity by inducing muscle mass [51]. In parallel with this information, the application of ETEBFR and ETEIN to HepG2-IR cells indicated that these compounds are candidates for natural phospho-NFκB inhibitors because they reduce the level of phospho-NFkB protein. 

As presented in Table 1, due to the high phytochemical content of ETE and its antioxidant capacity and AGE inhibition, the ROS balance, which was disrupted by reducing the level of phospho-NFκB in the molecular pathway, was restored. It has been revealed that ROS-induced insulin resistance can be eliminated by decreasing RAGE and AP-1 protein levels. It has been demonstrated that the increase in RAGE, the receptor of AGE, which occurs as a result of the irreversible reaction between reducing sugars and amino groups of proteins, lipids, and nucleic acids, is involved in the development of type 2 diabetes, hyperglycemia, and insulin resistance. It is known that the stress pathway is triggered in the endoplasmic reticulum by the binding of AGEs to the cell receptor RAGE, after which the expression of pro-inflammatory molecules such as interleukin-6 (IL-6) and tumor necrosis factor alpha (TNF-α) increases and ROS-induced diabetes develops. The AGE/RAGE interaction results in insulin metabolism impairment as well as glomerular hypertrophy and nephropathy [52]. The fact that the sub-fractions of ETEBFR and ETEIN obtained as a result of the gastric digestion model applied to ETE have AGE inhibition activity as well as the fact that the RAGE level decreased after these extracts were applied to HepG2-IR cells are shown in our results in Figure 3. These data show that healthy insulin metabolism may be restored by preventing the formation of the AGE/RAGE complex of ETE. On the other hand, the increased ROS level as a result of the AGE/RAGE interaction causes the activation of some transcription factors, such as activator protein-1 (AP-1) and cytokines, in the diabetes and inflammation pathways [53]. AP-1 activation, which is controlled by the mitogen-activated protein kinase (MAPK) and NFκB pathways, may lead to various diseases such as diabetic retinopathy, cancer, asthma, psoriasis, renal dysfunction, and liver disorders [54]. In accordance with this information, HepG2 cells have increased levels of phospho-NfκB and AP-1 proteins, which are over-synthesized after IR development, as described before, and play a crucial role in the development of diabetes. Studies supporting the findings in this study with other extracts have shown that after application, the levels of nuclear NfκB and AP-1 proteins in cells decrease [55]. After the administration of ETEBFR and ETEIN to the HepG2-IR cell model, significantly lower levels of the phospho-NFκB, RAGE, and AP-1 proteins were detected in these cells than in control cells. These results indicate that the ETE can be used to treat insulin resistance and diabetic kidney disorders in a gastric digestion model, which is consistent with ethnobotanical claims. It should be noted that the limited number of phenolics studied here can be regarded as a limitation to our study. In addition, confirming the results with in vivo studies may be the subject of the upcoming research.

## 4. Conclusions

Our present study investigated the benefits of ETE on diabetes and kidney disorders through various mechanisms at the cellular level. First, to increase the accuracy of the bioactivity assays by considering the possible pharmacokinetic effects on oral consumption, a human digestion simulation procedure was conducted on the ETE. To elucidate the relationship between chemical compounds and bioactivity, an extensive LC-MS/MS procedure was used on the extracts and fractions, together with in vitro phytochemical assays, and major phenolic compounds and their alterations were determined through simulated digestion. In vitro antioxidant screening was accomplished via several methods, supported by the DCFDA assay to evaluate antioxidant potential at the cellular level. Various enzyme inhibition assays related to ethnobotanical utilization were performed, and alterations in protein synthesis were investigated. This study confirmed that even after gastric digestion, the bioavailable part of ETE has significant potential for inhibiting various enzymes, including urease, α-amylase, α-glucosidase, and PTP1B; reducing AGE formation; and reducing the synthesis of proteins such as AP-1, NFκB, and RAGE, which are related to the pathogenesis of diabetes and kidney disorders. In conclusion, the present study supports the ethnobotanical use of *Equisetum telmateia* Ehrh. in traditional medicine in Türkiye. 

## Figures and Tables

**Table 1:** Phytochemicals of ETE and its fractions based on LC-MS/MS analysis.

No	Analytes	RT *^a^*	M.I. (m/z) *^b^*	F.I. (m/z) *^c^*	Ion. Mode	Amounts (µg/g)		
						*BFR*	*PG*	*IN*
1	Quinic acid	3.0	190.8	93.0	Neg	0.262	0.36	0.351
2	Fumaric aid	3.9	115.2	40.9	Neg	N.D.	N.D.	N.D.
3	Aconitic acid	4.0	172.8	129.0	Neg	0.232	0.377	0.224
4	Gallic acid	4.4	168.8	79.0	Neg	N.D.	N.D.	N.D.
5	Epigallocatechin	6.7	304.8	219.0	Neg	N.D.	N.D.	N.D.
6	Protocatechuic acid	6.8	152.8	108.0	Neg	1.17	1.48	1.18
7	Catechin	7.4	288.8	203.1	Neg	N.D.	N.D.	N.D.
8	Gentisic acid	8.3	152.8	109.0	Neg	N.D.	N.D.	N.D.
9	Chlorogenic acid	8.4	353.0	85.0	Neg	0.046	0.052	0.046
10	Protocatechuic aldehyde	8.5	137.2	92.0	Neg	0.676	1.06	0.90
11	Tannic acid	9.2	182.8	78.0	Neg	N.D.	N.D.	N.D.
12	Epigallocatechin gallate	9.4	457.0	305.1	Neg	N.D.	N.D.	N.D.
13	1,5-dicaffeoylquinic acid	9.8	515.0	191.0	Neg	N.D.	N.D.	N.D.
14	4-OH Benzoic acid	10.5	137.2	65.0	Neg	N.D.	N.D.	N.D.
15	Epicatechin	11.6	289.0	203.0	Neg	N.D.	5.63	1.13
16	Vanilic acid	11.8	166.8	108.0	Neg	N.D.	N.D.	N.D.
17	Caffeic acid	12.1	179.0	134.0	Neg	0.099	0.14	0.097
18	Syringic acid	12.6	196.8	166.9	Neg	N.D.	N.D.	N.D.
19	Vanillin	13.9	153.1	125.0	Poz	N.D.	N.D.	N.D.
20	Syringic aldehyde	14.6	181.0	151.1	Neg	N.D.	N.D.	N.D.
21	Daidzin	15.2	417.1	199.0	Poz	N.D.	N.D.	N.D.
22	Epicatechin gallate	15.5	441.0	289.0	Neg	N.D.	N.D.	N.D.
23	Piceid	17.2	391.0	135/106.9	Poz	N.D.	N.D.	N.D.
24	*p*-Coumaric acid	17.8	163.0	93.0	Neg	N.D.	N.D.	N.D.
25	Ferulic acid-D3-IS *^h^*	18.8	196.2	152.1	Neg	N.A.	N.A.	N.A.
26	Ferulic acid	18.8	192.8	149.0	Neg	N.D.	N.D.	N.D.
27	Sinapic acid	18.9	222.8	193.0	Neg	N.D.	N.D.	N.D.
28	Coumarin	20.9	146.9	103.1	Poz	0.064	0.083	0.052
29	Salicylic acid	21.8	137.2	65.0	Neg	N.D.	N.D.	N.D.
30	Luteolin 7-O-β-D-glucoside	23.7	447.0	284.0	Neg	0.187	0.167	0.053
31	Quercetin 3-glucuronide	24.1	477.0	150.9	Neg	N.D.	N.D.	N.D.
32	Rutin-D3-IS *^h^*	25.5	612.2	304.1	Neg	N.A.	N.A.	N.A.
33	Rutin	25.6	608.9	301.0	Neg	0.083	0.102	0.076
34	Isoquercitrin	25.6	463.0	271.0	Neg	0.062	0.077	0.048
35	Hesperidin	25.8	611.2	449.0	Poz	0.033	0.041	0.024
36	*o*-Coumaric acid	26.1	162.8	93.0	Neg	N.D.	N.D.	N.D.
37	Genistin	26.3	431.0	239.0	Neg	N.D.	N.D.	N.D.
38	Rosmarinic acid	26.6	359.0	197.0	Neg	N.D.	N.D.	N.D.
39	Ellagic acid	27.6	301.0	284.0	Neg	N.D.	N.D.	N.D.
40	Apigenin 7-glucoside	28.2	431.0	269.0	Neg	0.035	0.026	0.013
41	Quercitrin	29.8	447.0	301.0	Neg	N.D.	N.D.	N.D.
42	Kaempferol 3-O-glucoside	30.4	447.0	255.0	Neg	3.11	3.49	1.70
43	Kaempferol-3-O-rutinoside	30.6	592.9	255.0/284.0	Neg	7.02	8.41	5.34
44	Fisetin	30.6	285.0	163.0	Neg	N.D.	N.D.	N.D.
45	Daidzein	34.0	253.0	223.0	Neg	N.D.	N.D.	0.173
46	Quercetin-D3-IS *^h^*	35.6	304.0	275.9	Neg	N.A.	N.A.	N.A.
47	Quercetin	35.7	301.0	272.9	Neg	0.036	N.D.	N.D.
48	Naringenin	35.9	270.9	119.0	Neg	0.242	0.107	0.036
49	Hesperetin	36.7	301.0	136.0/286.0	Neg	N.D.	N.D.	N.D.
50	Luteolin	36.7	284.8	151.0/175.0	Neg	0.098	0.033	N.D.
51	Genistein	36.9	269.0	135.0	Neg	N.D.	N.D.	0.003
52	Kaempferol	37.9	285.0	239.0	Neg	1.285	0.258	0.02
53	Apigenin	38.2	268.8	151.0/149.0	Neg	0.049	0.008	N.D.
54	Amentoflavone	39.7	537.0	417.0	Neg	N.D.	N.D.	N.D.
55	Chrysin	40.5	252.8	145.0/119.0	Neg	N.D.	N.D.	N.D.
56	Acacetin	40.7	283.0	239.0	Neg	0.026	N.D.	N.D.

*^a^* N.D.: not detected; N.A.: not applicable; IS: internal standard; RT: retention time; *^b^* MI: multiple ion; *^c^* FI: fragment ion; *^h^*: internal standards

**Table 2:** Results of in vitro evaluation for phytochemical profile.

	ETE BFR ^A^	ETE PG	ETE IN
TPC ^BC^*	300 ± 7 ^a^	263 ± 11 ^b^	190 ± 5 ^c^
TFC ^D^	93.7 ± 0.3 ^a^	21.3 ± 0.1 ^b^	7.7 ± 0.01 ^c^

^A^ Abbreviations = BFR: non-digested; PG: post-gastric; IN: bioavailable. ^B^ Abbreviations = TPC: total phenolic content; TFC: total flavonoid content. ^C^ Results are expressed as the mean of triplicates ± standard deviation (S.D.) and as mg gallic acid equivalents (GAE) in 1 g sample. ^D^ Results are expressed as the mean of triplicates ± standard deviation (S.D.) and as mg quercetin equivalents (QE) in 1 g sample. * Different lowercase letters in the same row indicate significance (*p* < 0.05).

**Table 3:** In vitro preliminary tests for the antioxidant potential.

	ETE BFR ^A^	ETE PG	ETE IN
DPPH ^BC^*	272 ± 1 ^a^	140 ± 5 ^b^	72 ± 3 ^c^
TOAC ^D^	852 ± 15 ^a^	398 ± 11 ^b^	329 ± 18 ^c^
FRAP ^E^	1.82 ± 0.14 ^a^	1.23 ± 0.04 ^b^	0.37 ± 0.03 ^c^
CUPRAC ^D^	387 ± 12 ^a^	253 ± 3 ^b^	86 ± 2 ^c^

^A^ Abbreviations = BFR: non-digested; PG: post-gastric; IN: bioavailable. ^B^ Abbreviations = DPPH: 2,2-diphenyl-1,1-picrylhydrazyl; TOAC: total antioxidant capacity; FRAP: ferric reducing antioxidant power; CUPRAC: cupric reducing antioxidant capacity. ^C^ Results are expressed as the mean of triplicates ± standard deviation (S.D.) and as mg BHT equivalents (BHTE) in 1 g sample. ^D^ Results are expressed as the mean of triplicates ± standard deviation (S.D.) and as mg ascorbic acid (AAE) in 1 g sample. ^E^ Results are expressed as the mean of triplicates ± standard deviation (S.D.) and as mM FeSO_4_ equivalents in 1 g sample. * Different lowercase letters in the same row indicate significance (*p* < 0.05).

**Table 4:** In vitro enzyme and AGE inhibition results.

	ETE BFR ^AB^	ETE PG	ETE IN	STD ^C^
Urease ^C^*	92.5 ± 1.9 ^a^	78.8 ± 1.6 ^b^	87.3 ± 1.6 ^c^	99.1 ± 0.2 ^d^
α-Amylase	76.6 ± 2.2 ^a^	46.4 ± 0.9 ^b^	60.9 ± 0.9 ^c^	94.6 ± 0.9 ^d^
α-Glucosidase	82.6 ± 2.0 ^a^	55.9 ± 0.3 ^b^	71.6 ± 2.1 ^c^	90.8 ± 1.0 ^d^
PTP1B ^D^	79.0 ± 1.4 ^a^	61.8 ± 1.4 ^b^	66.8 ± 1.9 ^c^	96.7 ± 2.1 ^d^
AGE	87.6 ± 3.6 ^a^	62.2 ± 1.6 ^b^	71.5 ± 0.9 ^c^	95.3 ± 2.9 ^d^

^A^ Abbreviations = BFR: non-digested; PG: post-gastric; IN: bioavailable. ^B^ All results are given as percent inhibition ± standard deviation. ^C^ All standard substances and samples were used at 1 mg/mL concentration. Standard substances: thiourea for urease assay, acarbose for α-amylase assay, quercetin for α-glucosidase and AGE assay, and ursolic acid for PTP1B assay. ^D^ PTP1B: Protein tyrosine phosphatase 1B; AGE: advanced glycation end product. * Different lowercase letters in the same row indicate significance (*p* < 0.05).

## Data Availability

The original contributions presented in the study are included in the article, further inquiries can be directed to the corresponding author.
